# Virome Profiling of Bats from Myanmar by Metagenomic Analysis of Tissue Samples Reveals More Novel Mammalian Viruses

**DOI:** 10.1371/journal.pone.0061950

**Published:** 2013-04-22

**Authors:** Biao He, Zuosheng Li, Fanli Yang, Junfeng Zheng, Ye Feng, Huancheng Guo, Yingying Li, Yiyin Wang, Nan Su, Fuqiang Zhang, Quanshui Fan, Changchun Tu

**Affiliations:** 1 Key Laboratory of Jilin Province for Zoonosis Prevention and Control, Institute of Military Veterinary, Academy of Military Medical Sciences, Changchun, Jilin, China; 2 Center for Disease Control and Prevention, Chengdu Military Region of the People’s Liberation Army, Kunming, Yunnan, China; The University of Hong Kong, Hong Kong

## Abstract

Bats are reservoir animals harboring many important pathogenic viruses and with the capability of transmitting these to humans and other animals. To establish an effective surveillance to monitor transboundary spread of bat viruses between Myanmar and China, complete organs from the thorax and abdomen from 853 bats of six species from two Myanmar counties close to Yunnan province, China, were collected and tested for their virome through metagenomics by Solexa sequencing and bioinformatic analysis. In total, 3,742,314 reads of 114 bases were generated, and over 86% were assembled into 1,649,512 contigs with an average length of 114 bp, of which 26,698 (2%) contigs were recognizable viral sequences belonging to 24 viral families. Of the viral contigs 45% (12,086/26,698) were related to vertebrate viruses, 28% (7,443/26,698) to insect viruses, 27% (7,074/26,698) to phages and 95 contigs to plant viruses. The metagenomic results were confirmed by PCR of selected viruses in all bat samples followed by phylogenetic analysis, which has led to the discovery of some novel bat viruses of the genera *Mamastrovirus, Bocavirus*, *Circovirus*, *Iflavirus* and *Orthohepadnavirus* and to their prevalence rates in two bat species. In conclusion, the present study aims to present the bat virome in Myanmar, and the results obtained further expand the spectrum of viruses harbored by bats.

## Introduction

Bats, comprising the second largest mammalian population in the world and distributed globally with the exception of the two polar areas, belong to the order *Chiroptera* with 17 families and 925 species [1]. Bats are important virus reservoir animals and more than 60 viruses have been identified in them with many highly pathogenic to humans [2], including henipaviruses, Ebola virus, Marburg virus, dengue virus, lyssaviruses and SARS-like coronavirus [3]–[7]. Most recently, Bokeloh and Shimoni bat viruses, circovirus, bocavirus, retrovirus, astrovirus, and Cedar virus have been identified as new bat viruses with some never having been reported in other animals, suggesting that bats could be a large virus bank and breeding ground for viruses [4], [8]–[15]. In China, viruses are increasingly being detected in, or isolated from bats, such as coronavirus, circovirus, astrovirus, Xi River virus, Japanese encephalitis virus (JEV), Chikungunya virus, Tuhoko virus, picornavirus, adeno-associated virus and adenovirus [5], [9], [14], [16]–[22]. Notably, SARS coronavirus, which has infected more than 8,000 people and killed almost 800 worldwide, has been identified as likely originating from horseshoe bats in China [5], [23], [24]. However, all available studies so far fail to provide a complete understanding of the pathogen ecology of bat populations.

To control the outbreaks of emerging or re-emerging viral diseases and prevent the transmission of viruses from wildlife, particularly bats, to humans, monitoring the virus infection situation in natural hosts and vector animals is important. Availability of next generation sequencing-based viral metagenomics in recent years has provided a powerful tool for large-scale detection of known and unknown viruses existing in host animals [25], [26]. This new technology has been employed to explore the constitution of viral communities in such environments as oceans, lakes, various tissues, guts and feces of animals including bats has undoubtedly opened a new window to an understanding of the virus diversity in these environments and has provided a successful paradigm for future rapid discovery of new viruses in nature [10], [27]–[37].

The region covering Southeast Asia and Southern China is a main epicenter of emerging or re-emerging viral diseases due to its high human population, inadequately developed public health systems, and abundant and diverse wild animal resources with their illegal trading. Yunnan province in China is a main and busy trading route between Southeast Asia and China and shares a long border with Myanmar, Laos and Vietnam. Studies have shown that many viruses, including Nipah virus, JEV, Chikungunya virus, and circovirus, are present in bats in Cambodia, Thailand and Yunnan [9], [17], [18], [35], [38]–[40]. To expand these studies to Myanmar, we applied viral metagenomics to determine the virome of bats collected from areas of Myanmar adjoining Yunnan. Results revealed 24 virus families in these bats, including phages and viruses of plants and insects as well as vertebrates. Several viruses of the genera *Orthohepadnavirus, Mastadenovirus, Mamastrovirus,* and *Bocavirus*, have been characterized as new viruses. This work has expanded our knowledge of bat viruses and their geodistribution in Southeast Asia and could be helpful in establishing effective surveillance of wildlife-associate zoonoses.

## Materials and Methods

### Bat Collection

A total of 853 freshly dead insectivorous bats comprising Miniopterus fuliginosus (M. fuliginosus), Hipposideros armiger (H. armiger), Rhinolophus ferrumequinum (R. ferrumequinum), Myotis chinensis (M. chinensis), Megaderma lyra (M. lyra) and Hipposideros fulvus (H. fulvus) species, were purchased in October and December, 2008, from Burmese living in border areas of Myanmar adjoining Yunnan province. All bats were collected from habitats in two Myanmar counties, Sedon and Wutao, which are close to Tengchong county of Yunnan. The organs of the thorax and abdomen of each bat were separately collected and stored at −20°C. The predominant species sampled was M. fuliginosus and all bat samples were separated into the following 4 groups based on their location and species: XM (M. fuliginosus from Sedon), XO (the remaining 3 species from Sedon), WM (M. fuliginosus from Wutao) and WO (the remaining 5 species from Wutao) (table 1). The collection and sampling of bats for this study was approved by the Administrative Committee on Animal Welfare of the Institute of Military Veterinary, Academy of Military Medical Sciences, China.

**Table 1:** Bat species tested in the metagenomic analysis and validation summary of selected viruses^a^.

Virus	Sedon				Wutao					
	M. F.	R. F.	H. A.	M. C.	M. F.	R. F.	H. A.	M. C.	M. L.	H. F.
	n = 320	n = 92	n = 7	n = 1	n = 320	n = 84	n = 1	n = 10	n = 6	n = 12
AstV	12(4%)	0	0	0	0	2(2%)	0	0	0	0
IfV	26(8%)	0	0	0	56(18%)	0	0	0	0	0
CV	0	6(7%)	0	0	0	0	0	0	0	0
AdV	0	0	0	0	0	1(1%)	0	0	0	0
AAV	0	0	0	0	0	1(1%)	0	0	0	0
BoV	6(2%)	0	0	0	20(6%)	0	0	0	0	0

a M. F. : *M. fuliginosus*; R. F. : *R. ferrumequinum*; H. A.: *H. armiger*; M. C.: *M. chinensis*; M. L.: *M. lyra*; H. F.: *H. fulvus*; AstV: astrovirus; IfV: iflavirus; CV: circovirus; AdV: adenovirus; AAV: adeno-associated virus; BoV: bocavirus.

### Preparation of Tissue Samples and Extraction of Viral Nucleic Acids

Briefly, bat organs and tissues (laryngopharynx, trachea, lung, heart, liver, spleen, stomach, gut, kidney and bladder) of each of the 4 groups were pooled and homogenized in a blender (Waring, Torrington, CT) with SM buffer (50 mM Tris, 10 mM MgSO_4_, 0.1 M NaCl, pH7.5). Following centrifugation at 8000×*g* at 4°C for 30 min to remove cell debris and foreign materials, the supernatants were immediately filtered through 0.45-µm and 0.22-µm Pellicon II filters (Millipore, Billerica, MA). Filtrates were then concentrated in a 100-kDa Pellicon II filter (Millipore), and filtered once again through 0.22-µm syringe filters (Millipore). To eliminate contamination by host genome and other free nucleic acids, 14 U Turbo DNase (Ambion, Austin, TX), 25 U Benzonase Nuclease (Novagen, San Diego, CA) and 20 U RNase I (Fermentas, Ontario, Canada), ddH_2_O and 10×DNase buffer (Ambion) were added to 116 µl filtrate to a final volume of 140 µl followed by digestion at 37°C for 1 h. The viral RNAs and DNAs were then extracted immediately using TRIzol (TaKaRa, Dalian, China) according to the manufacturer’s protocol. Total viral nucleic acids were dissolved in RNase-free H_2_O (TaKaRa) and used immediately for the following reverse transcription.

### Reverse Transcription with Anchored Random Primers

The above viral nucleic acids were reverse transcribed with SuperScript III reverse transcriptase (Invitrogen, Carlsbad, CA) using a published method [36]. Briefly, four 20-nucleotide barcode DNAs were designed online (http://www.changbioscience.com/primo/primor.html) and anchored to random hexamers to prepare barcode primers. Each of the four groups of nucleic acids was added separately to the four barcode primers (10 µM). Mixtures were heated at 65°C for 5 min and cooled on ice for 2 min to denature the secondary structure. Then 4 µl 5×first-strand buffer, 1 µl 0.1 M dithiothreitol (DTT), 1 µl 40 U RNase OUT, 1 µl 10 mM deoxynucleoside triphosphates (dNTPs) and 1 µl 200 U SuperScript III reverse transcriptase were added along with RNase-free H_2_O to bring the final volumes to 20 µl. The reactions were incubated at 25°C for 5 min followed by 50°C for 60 minutes, then inactivated at 75°C for 15 min.

### Double Stranded cDNA (dscDNA) Synthesis with Klenow Fragment

To synthesize dscDNA, a 3′–5′exo^–^ Klenow fragment (5 U; New England Biolabs, Beijing, China) was added to the cDNA mixture and barcode primers, then incubated at 37°C for 60 min, after which the enzyme was inactivated at 75°C for 10 min. To remove phosphates and free single-stranded bases in the dscDNA reaction, 2 U shrimp alkaline phosphatase (SAP, TaKaRa) and 2.5 U exonuclease I (TaKaRa) were added to the dscDNA reaction mixture along with 10×SAP buffer and ddH_2_O to a final volume of 50 µl, then incubated at 37°C for 60 min and inactivated at 75°C for 10 min.

### Sequence-independent Single Primer Amplification (SISPA) and Purification of PCR Products

To obtain sufficient viral nucleic acid, SISPA was employed to amplify the dscDNA with the Accuprime Taq DNA Polymerase System (Invitrogen) according to the manufacturer’s protocol. Briefly, a 50 µl reaction system containing 10 µl of the above dscDNA mixture, barcode DNA as primer (20 µM), 10×Accuprime buffer I, Accuprime Taq DNA Polymerase (1 U) and ddH_2_O was denatured at 94°C for 2 min, followed by 40 cycles of 94°C denaturing for 30 s, 55°C annealing for 30 s, 68°C extending for 1 min with final 68°C extension for 8 min. The PCR products were then purified using the QIAquick PCR Purification Kit (Qiagen, Hilden, Germany) and dissolved in 50 µl TE buffer (100 mM Tris-HCl, 10 mM EDTA, pH8.0).

### High-throughput Sequencing

The above purified PCR products of the four groups were pooled together and then subjected to Solexa sequencing in one lane by the Beijing Genome Institute (BGI, Shenzhen, China). Briefly, the pooled purified PCR products were ultrasonicated to produce DNA fragments of about 180 bp, and then treated with Klenow and dATP to generate 3′-dA overhangs. After ligation of the fragments to Solexa adaptors, the DNAs were subjected to PCR with the adaptor primers to construct a genomic DNA library. The amplicons were bound to a flow cell to which was then added fluorescent-labeled dNTPs. The DNA sequences were obtained by the mechanism of Sequencing-by-Synthesis (SBS; Illumina). Base calling was conducted with the program of GAPipeline using default settings. After removing the adaptor sequences and no-calling reads, the sequences of the four groups were differentiated by their barcodes and then assembled into contigs with SOAPdenove software (BGI, Shenzhen, China). Contigs and sequences longer than 100 bp were defined as significant data for further *in silico* analysis.

### *In silico* Analysis of Contigs

The significant data were aligned online against the nonredundant and viral reference databases of GenBank with BLASTx and BLASTn. The BLAST hits were defined as significant if E value ≤10e−5 [10]. Contigs of bacteria and eukaryotes were eliminated and virus-like sequences were subjected to further analysis.

### Confirmation of Selected Viruses

To validate the results of Solexa sequencing, primers to amplify identified viral sequences were synthesized according to publications, or designed based on the Solexa sequences obtained in this study using Primer 5 (Premier Biosoft International, Palo Alto, CA) and Genefisher (http://bibiserv.techfak.uni-bielefeld.de/bibi/Tools.html). Primer sequences used in the validation are available upon request. Viral RNA and DNA were automatically extracted with RNeasy and QIAamp DNA mini kits respectively (Qiagen, Hilden, Germany) in a QIAcube (Qiagen) from organ tissues of each bat and subjected to all nucleic acid amplification tests. Viral RNA was amplified using RT-PCR or RT-nested PCR, while viral DNA was subjected directly to PCR with negative but no positive control to avoid false positive results. The amplification of nucleic acids was conducted with PCR Master Mix (Tiangen, Beijing, China) according to the manufacturer’s protocol. Positive PCR products were sequenced in both directions commercially by an ABI 3730 DNA Analyzer (Invitrogen, Beijing, China). Two near full genomes of bat bocaviruses were constructed with a genome walking kit (TaKaRa, Dalian, China) following the manufacturer’s protocol.

### Phylogenetic Analysis

Alignments of nucleotide (nt) and amino acid (aa) sequences with other representatives of known viruses were conducted using Clustal W version 2.0. (Accession numbers are shown in the trees). Phylogenetic trees were generated by the neighbor-joining method of MEGA 5 with 1,000 bootstrap replicates [41]. ORFs were identified by NCBI ORF Finder (http://www.ncbi.nlm.nih.gov/gorf/gorf.html); the secondary structure of circovirus genome was predicted with the mfold Web Server (http://mfold.rna.albany.edu/?q=mfold).

### Nucleotide Sequence Accession Numbers

The data from Solexa sequencing have been deposited in the GenBank Sequence Reads Archive under accession numbers SRA059263 and SRA05929. All partial and full genome sequences of selected viruses have been deposited in GenBank under accession numbers JX863704 to JX863737 and KC339249 to KC339251.

## Results

### Solexa Sequencing and General Virome of the Bats

After removal of barcode and host gene sequences, 3,742,314 reads were obtained by Solexa sequencing with 88% of reads overlapping into 1,649,512 contigs with an average length of 114 nt. As shown in table 2, of 1, 649,512 contigs 33,501 (2%) and 19,940 (1%) were annotated to eukaryotes and bacteria respectively, while 1,569,373 (95%) were classified as unknown sequences. The remaining 26,698 (2%) contigs were matched to viruses according to the defined cutoff value. All viral sequences could be classified into 24 viral families: 13 dsDNA, 2 ssDNA, 2 retro-transcribing, 1 ssRNA minus stranded, 5 ssRNA plus stranded and 1 dsRNA. The results summarized in table 3 show that the virome of the bats in the study comprised viruses of vertebrates, insects, plants and bacteria, with vertebrate virus-like contigs as the predominant sequences accounting for 45% (12,086/26,698). Sequences relating to insect viruses were the 2^nd^ most abundant, constituting 28% (7,443/26,698), followed by phage at 27% (7,074/26,698) of the total viral contigs, while only 95 contigs were related to plant viruses.

**Table 2:** Overview of Solexa sequencing^a^.

Pool	Datasize(nt)	Reads	Contigs	Rate	A.length	Eukaryotic	Bacterial	Virus-like	Unknown
XM	28,619,736	555,398	240,886	87%	119	8,845	374	2,321	229,346
XO	46,278,083	868,378	396,140	91%	117	3,628	1,363	3,076	388,073
WM	62,700,753	1,287,416	554,521	86%	113	3,628	770	17,336	532,787
WO	49,579,217	1,031,122	457,965	89%	108	17,400	17,433	3,965	419,167
Total	187,177,789	3,742,314	1,649,512	88%	114	33,501	19,940	26,698	1,569,373

a XM: *M. fuliginosus* from Sedon County; XO: other species bats from Sedon County; WM: *M. fuliginosus* from Wutao County; WO: other species bats from Wutao County; Datasize: the total length of contigs; Reads: the quantity of total reads after removal of contamination by host genome; Contigs: the numbers of contigs after overlapping; Rate: the reads ratio between the overlapped and the total; A. length: the average length of contigs; Eukaryotic, Bacterial and Virus-like: contigs relating to eukaryotes, bacteria and viruses; Unknown: sequences without a hit in the GenBank nonredundant database.

**Table 3:** Overview of viral contigs.

Viral host	Viral family	Viral genus	XM	XO	WM	WO	Total
Vertebrate	*Adenoviridae*	*Mastadenovirus*	0	0	0	1	1
	*Herpesviridae*	*Cytomegalovirus*	9	33	79	53	174
		*Mardivirus*	0	3	0	2	5
		*Roseolovirus*	4	5	2	6	17
	*Alloherpesviridae*	*Ictalurivirus*	20	2	333	47	402
	*Papillomaviridae*	*Alphapapillomavirus*	9	0	1	7	17
	*Polyomaviridae*	*Polyomavirus*	10	2	1	6	19
	*Picobirnaviridae*	*Picobirnavirus*	0	0	0	1	1
	*Hepadnaviridae*	*Orthohepadnavirus*	64	10	11,147	75	11,296
	*Circoviridae*	*Circovirus*	0	17	0	0	17
	*Poxviridae*	*Orthopoxvirus*	11	5	12	0	28
	*Retroviridae*	*Deltaretrovirus*	0	22	0	1	23
	*Parvoviridae*	*Dependovirus*	0	0	0	4	4
		*Bocavirus*	9	0	11	0	20
	*Astroviridae*	*Mamastrovirus*	35	0	0	22	57
	*Picornaviridae*	*Kobuvirus*	1	0	1	1	3
		*Enterovirus*	0	1	0	0	1
	*Flaviviridae*	*Hepacivirus*	0	0	0	1	1
		Subtotal	172	100	11,587	227	12,086
Insect	*Baculoviridae*	*Alphabaculovirus*	0	0	0	1	1
		*Betabaculovirus*	57	2,674	42	2201	4,974
	*Iridoviridae*	*Ictalurivirus*	0	0	0	1	1
	*Polydnaviridae*	*Bracovirus*	14	14	22	59	109
		*Ichnovirus*	1,779	0	42	29	1,850
	*Parvoviridae*	*Densovirus*	0	22	1	3	26
		*Brevidensovirus*	2	0	3	0	5
	*Iflaviridae*	*Iflavirus*	191	0	286	0	477
		Subtotal	2,043	2,710	396	2,294	7,443
Plant	*Phycodnaviridae*	*Chlorovirus*	0	0	1	1	2
		*Coccolithovirus*	4	0	22	4	30
	*Bunyaviridae*	*Tospovirus*	0	0	0	1	1
	*Bromoviridae*	*Cucumovirus*	0	29	0	33	62
		Subtotal	4	29	23	39	95
Bacteria	*Myoviridae*	*P1-like virus*	1	0	59	2	62
		*P2-like virus*	0	2	65	1	68
		*SPO1-like virus*	2	1	2,218	98	2,319
		*Mu-like virus*	0	0	5	0	5
		*T4-like virus*	35	5	1,930	1	1,971
	*Podoviridae*	*Epsilon15-like virus*	5	0	13	32	50
		*Phieco32-like virus*	0	0	13	0	13
		*N4-like virus*	0	0	1	0	1
		*P22-like virus*	5	0	10	9	24
		*SP6-like virus*	0	0	53	29	82
		*T7-like virus*	39	220	825	1,149	2,233
	*Siphoviridae*	*C2-like virus*	0	0	1	2	3
		*lambda-like virus*	2	0	3	1	6
		*T1-like virus*	12	9	131	81	233
		*T5-like virus*	1	0	3	0	4
		Subtotal	102	237	5,330	1,405	7,074
		Total	2,321	3,076	17,336	3,965	26,698

### Sequences Relating to Vertebrate Viruses

As shown in table 3, the contigs of vertebrate viruses comprised the largest virus population in the virome of all four groups, and could be classified into 14 families: *Adenoviridae*, *Alloherpesviridae*, *Herpesviridae*, *Papillomaviridae*, *Polyomaviridae*, *Poxviridae*, *Picobirnaviridae*, *Hepadnaviridae*, *Retroviridae*, *Circoviridae*, *Parvoviridae* (*Parvovirinae*), *Astroviridae*, *Flaviviridae* and *Picornaviridae*. Members of the *Alloherpesviridae*, *Herpesviridae*, *Hepadnaviridae*, *Papillomaviridae*, *Parvovirinae*, *Polyomaviridae*, *Poxviridae and Picornaviridae* were likely common viruses harbored by bats since at least 3 of the 4 groups contained their sequences in viral contigs. These contigs relating to mastadenovirus, circovirus, dependovirus, bocavirus, parvovirus, mamastrovirus, kobuvirus, iflavirus, had strong BLAST bit to viruses with bit score >200 and e value <10e−20. Notably, there were >10 thousand contigs relating to Hepadnaviridae, which covered 43% full genome and shared >70% nt identity with woodchuck hepatitis virus (WHV) and human hepatitis B virus (HBV). Herpesvirus-like contigs had diverse nt identities to known viruses, the longest reads with 210 nt sharing the highest nt identity of 70% with human herpesvirus 6B (data not shown). In the present study, ictalurivirus-, poxvirus-, polyomavirus-, papillomavirus- and hepacivirus-like sequences were not only annotated to viruses but also share nt/aa identities with some cellular organisms, probably as a result of integration of viral genes into their hosts or host genes into viral genomes since large DNA viruses have ability to acquire cellular genes over the course of their evolution [42]–[45]. Therefore, to confirm the presence of above large DNA viruses PCR detection and sequencing of longer fragments at multiple viral genomic regions is needed in the future.

### Sequences Relating to Insect Viruses, Phage and Plant Viruses

Sequences relating to insect viruses comprised the second largest proportion within the bat virome, and could be classified into five families: *Baculoviridae*, *Polydnaviridae*, *Iridoviridae*, *Iflaviridae* and *Parvoviridae* (*Densovirinae*) (Table 3). Viruses of the *Baculoviridae, Densovirinae* and *Polydnaviridae* were present in all four groups of bats. It is interesting to note that the iflavirus was present in, and *Iridoviridae* absent from, *M. fuliginosus* of both counties. Among the insect viruses detected, alpha- and betabaculoviruses within the *Baculoviridae*, and bracovirus and ichnovirus within the *Polydnaviridae* were first time discoveries in bats. Contigs of densovirus and iflavirus, found in bat guano from North America and China [35], [37], were also present in our study. Contigs of iflavirus identified here had >80% nt identity with the *Perina nuda* virus of genus *Iflavirus*.

According to previous studies, phage comprise a large fraction of the viromes of human, equine and bat fecal samples [35], [37], [46], [47]. For example, the sequences of phage in bat guano from California and Texas respectively accounted for 4% and 0.1% of the total reads [37], while phage sequences constituted >60% of the total in previously reported bat fecal samples in China [35]. In the present study, three phage families, *Myoviridae*, *Podoviridae* and *Siphoviridae*, were detected as being harbored by all four groups of bats, constituting 4% viral contigs in group XM, 2% in XO, 31% in WM and 1% in WO. In groups XM, XO and WO, the family *Podoviridae* was the most abundant, accounting for 48%, 93% and 87% of the total phage-like contigs respectively, while in WM the family *Myoviridae* was dominant, constituting 80% of phage-like contigs within the group. The wide prevalence of these three phage families in all 4 groups likely reflects the common infection status of phages in the gut bacterial microecosystems of bats in these areas.

Plants provide the most abundant viruses in human feces [34] and also exist commonly in bat guano, reflecting dietary traits [10], [35], [37]. In this study, 95 contigs of plant viruses were identified, comprising three families ― a more restricted number compared with the other virus groups. Cucumovirus within family *Bromoviridae*, tospovirus within family *Bunyaviridae*, and coccolithovirus and chlorovirus within family *Phycodnaviridae* were identified for the first time in bats.

### Confirmation of Metagenomic Results by PCR

To verify the results of the metagenomic analysis, specific primers of selected viruses (mainly from vertebrate viruses) were used to amplify the corresponding virus species from all bat samples with the following results.

### PCR Amplification of Astrovirus

**Figure 1:**
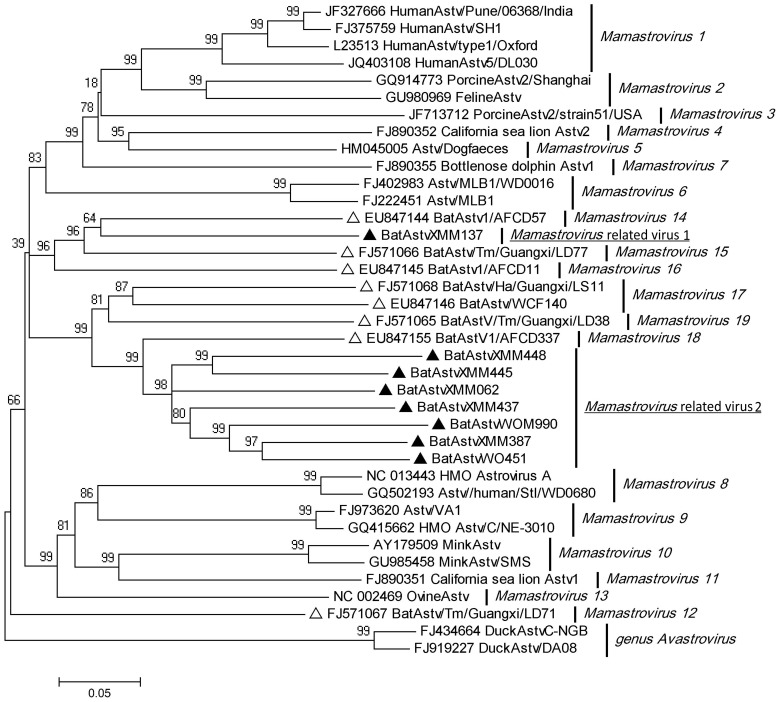
Phylogenetic analysis of bat astroviruses with representatives of all 19 species of mamastroviruses using 422 bp fragments of ORF1b gene. Sequences from our study are identified by filled triangles and from previous studies by open triangles.

The genus *Mamastrovirus*, belonging to the family *Astroviridae*, is composed of 19 viral species based on the ORF2 gene [48]. In our study 35 and 22 contigs relating to genus *Mamastrovirus* were obtained from groups XM and WO respectively (Table 3), but none from the other two groups. Testing of all bat lungs and guts by hemi-nested PCR using pan-bat astroviruses primers targeting a 422 nt fragment of ORF1b provided confirmation, resulting in 12 and 2 positive amplifications in guts of groups XM and WO respectively. The positive rate in *Miniopterus fuliginosus* was 4% (12/320). In group WO, only *Rhinolophus ferrumequinum* showed positive amplification in 2% (2/84) of samples, while all other bat species were negative. Of 15 amplicons, 8 (Accession numbers: JX863704–JX863711) were further sequenced and phylogenetically analyzed against representative astroviruses of all 19 species of *Mamastroviruses* based on the nt sequences. Results (Figure 1) showed that the taxonomic tree of the 19 species of *Mamastroviruses* based on a partial ORF1b gene was the same as that based on ORF2. Bat astroviruses detected in this study, however, could be further grouped into two branches with 57–63% nt identity between them. Of these, BatAstv XMM137 amplified from *M. fuliginosus* in county Sedon shared the highest identity of 69% with Mamastrovirus 14 found in bats from Hong Kong [12], and was likely a new Mamastrovirus species according to new ICTV criteria [48]. It was therefore tentatively classified as Mamastrovirus-related virus 1. The remaining 7 amplicons with 68–76% nt shared identity comprised a new group, and shared the highest nt identity (67–75%) with Mamastrovirus 18 found in Hong Kong bats [12]. These sequences constituted at least one new Mamastrovirus species according to new ICTV criteria, and were therefore classified as Mamastrovirus-related virus 2 [48].

### PCR Amplification of Iflaviruses

**Figure 2:**
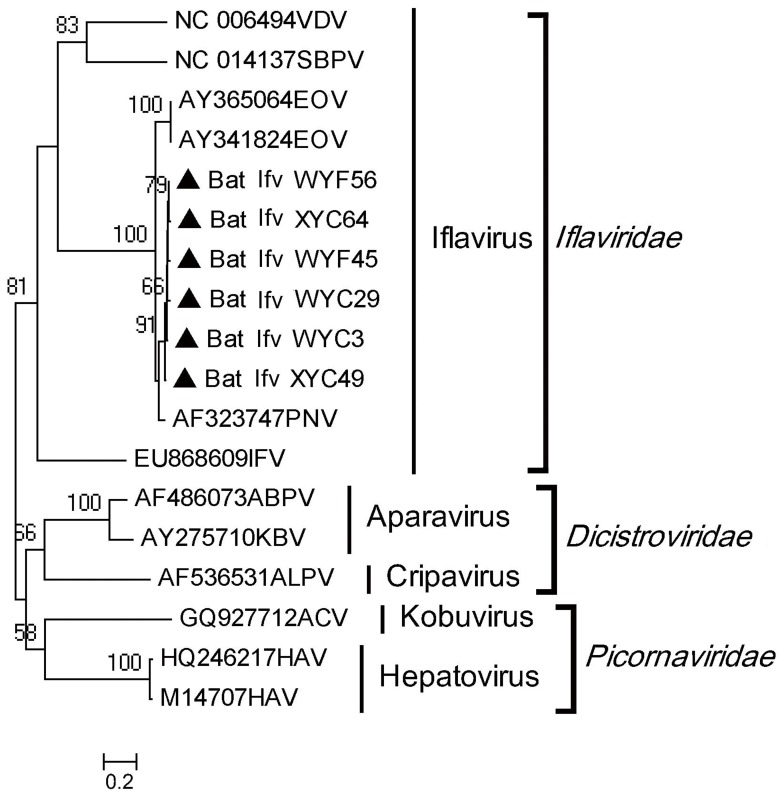
Phylogenetic analysis of partial ORF aa sequences deduced from 369 bp amplicons of bat iflaviruses with other representatives in the families of *Iflaviridae*, *Dicistroviridae* and *Picornaviridae*. The Sequences from our study are identified by filled triangles.

The family *Iflaviridae* has a single genus *Iflavirus* consisting of 7 viral species [48]. By Solexa sequencing, 191 and 286 contigs annotated to *Iflaviridae* were found in groups XM and WM respectively, showing high identity with *Ectropis obliqua* virus (EOV) and *Perina nuda* virus (PNV), both being insect viral species within genus *Iflavirus* (Table 3). Nested RT-PCR of all bat organs targeting 369 nt at the 3′ end of the single ORF confirmed the results of Solexa sequencing, and further showed that 18% (56/320) of guts of *M. fuliginosus* in WM and 8% (26/320) of *M. fuliginosus* in XM were positive for the genus *Iflavirus*. Sequences (Accession numbers: JX863712–JX863717) amplified from the two groups had >98% nt identity, indicating that the viruses from the two groups were the same variant, sharing 82% aa identity with PNV and 80% identity with EOV (Figure 2) [49].

### PCR Amplification of Bocavirus and Adeno-associated Virus

**Figure 3:**
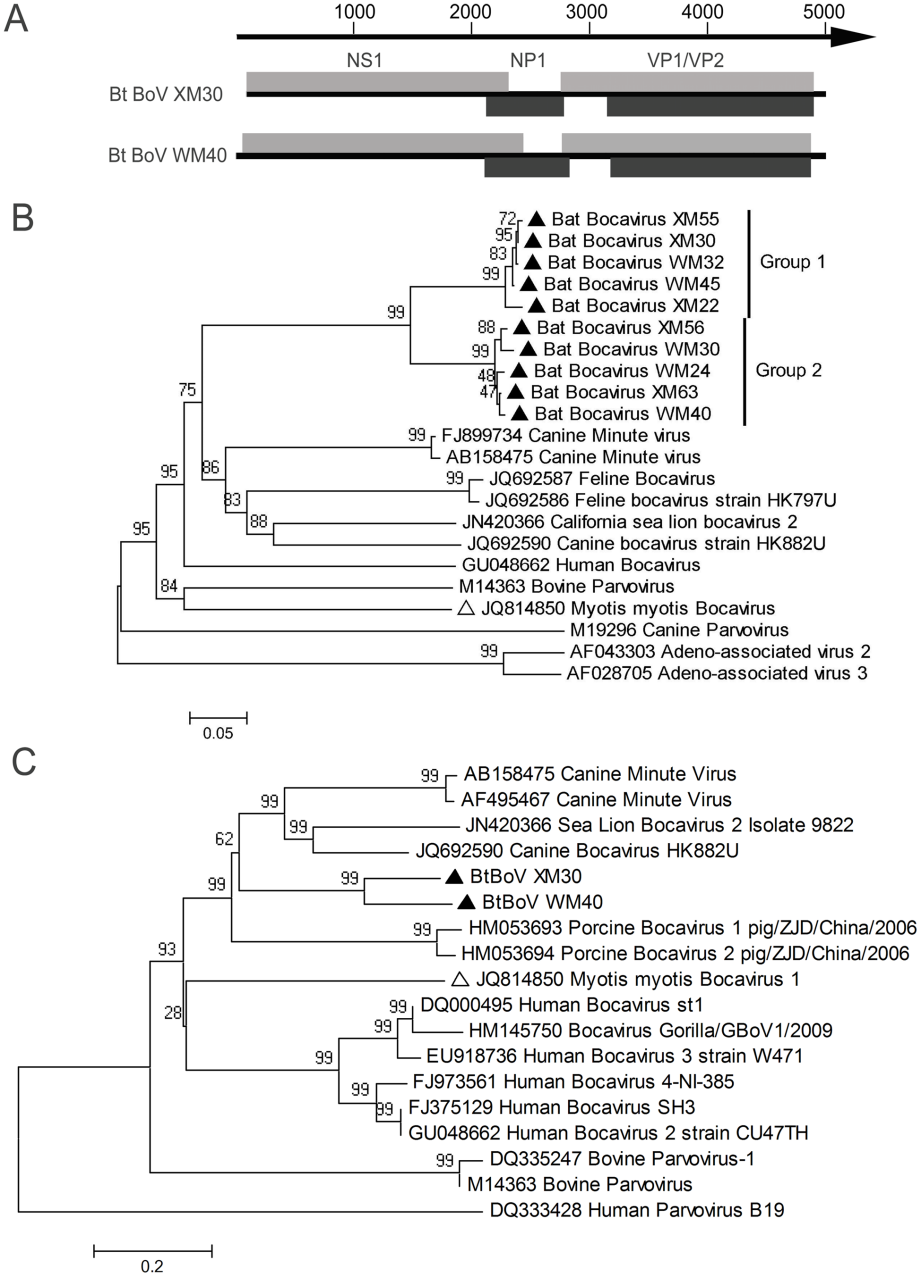
Phylogenetic analysis of BtBoVs. (A) Genome schematics of BtBoV XM30 and WM40; (B) Phylogenetic analysis of partial VP1 aa sequences deduced from 620 bp amplicons of bat bocaviruses and other representatives with two adeno-associated viruses as outgroup; (C) The phylogenetic tree of full NS1 aa sequences deduced from. Sequences from our study are identified by filled triangles and that of a bat bocavirus from a previous study by an open triangle.

By Solexa sequencing, 20 and 4 contigs were found closely related to bocavirus (BoV) and adeno-associated virus (AAV) respectively. In this viral metagenomic analysis, bocavirus sequence was detected in groups XM and WM, but not in XO and WO (Table 3). The finding by specific PCR of a partial VP1 ORF gene sequence using degenerated primers designed based on available bocavirus sequences in GenBank was consistent with the metagenomic analysis, and analysis of all bat samples further showed that 2% (6/320) of guts of *M. fuliginosus* in XM and 6% (20/320) of *M. fuliginosus* in WM were bocavirus positive (Table 1). Ten amplicons (five from each group, accession numbers: JX863718 - JX863727) with lengths of 620 nt were sequenced and phylogenetically analyzed against representative sequences of bocaviruses identified to date. Results showed that the newly identified bat bocaviruses were significantly divergent from known bocaviruses, with 39–53% aa identity. The highest aa identity (53%) observed was with canine minute virus and only 43% aa identity with *M. myotis* bocavirus, the first bat bocavirus found in China [10] (Figure 3B). Further phylogenetic analysis clustered our ten sequences into two independent groups. The highest amino acid identity between two groups was 85%, while that within a group was 97% (Figure 3B). This indicates the discovery of two new bocavirus species from bats. To further characterize the bocaviruses, nearly full length genomic sequences of Bt BoV XM30 in group 1 and Bt BoV WM40 in group 2 were obtained by gene walking. As shown in figure 3A, the genomes of Bt BoV XM30 with an incomplete 5′ end (accession number: KC339250) and Bt BoV WM40 (accession number: KC339251) were 4,832 and 4,995 nt containing four ORFs: NS1, NP1, VP1 and VP2. (Figure 3A). The aa sequences of NS1 were used to construct the phylogenetic tree with other representatives (Figure 3C). Multiple alignments showed that Bt BoV XM30 and WM40 shared 76% aa identity, with 35–54% aa and 44–56% nt identities with other bocaviruses. The highest (54%) aa identity was with canine bocavirus HK882U but only 40% to *Myotis myotis* bocavirus 1, with the same results obtained by analysis of VP1 (data not shown).

**Figure 4:**
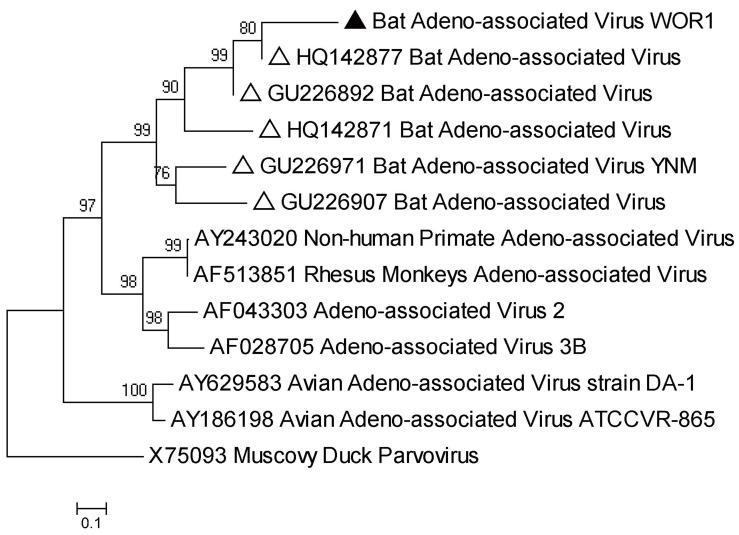
Phylogenetic analysis of 447 bp amplicons of partial VP gene sequence of bat adeno-associated viruses and other representatives with Muscovy duck parvovirus as outgroup. The sequences in our study are identified by filled triangles and those of bat bocavirus and adeno-associated viruses from previous studies by open triangles.

AAV is a member of the *Dependovirus* genus, *Parvovirinae* subfamily, and was first identified in the 1960s as co-existing with adenovirus without which it cannot replicate [50]. This virus has been found in bats by viral metagenomics in California and China [35], [37]. Here, AAV sequences were detected only in *R. ferrumequinum* of group WO (Table 3). An amplicon (accession number: JX863728) with 447 bp nucleotides of AAV targeting a partial VP region was obtained from this species and was phylogenetically grouped with other AAVs, showing 81% nt identity with bat AAV isolate 1715-HB-Rs-B (HQ142877) from a bat in Hubei province, China (Figure 4) [21].

### PCR Amplification of Adenovirus

**Figure 5:**
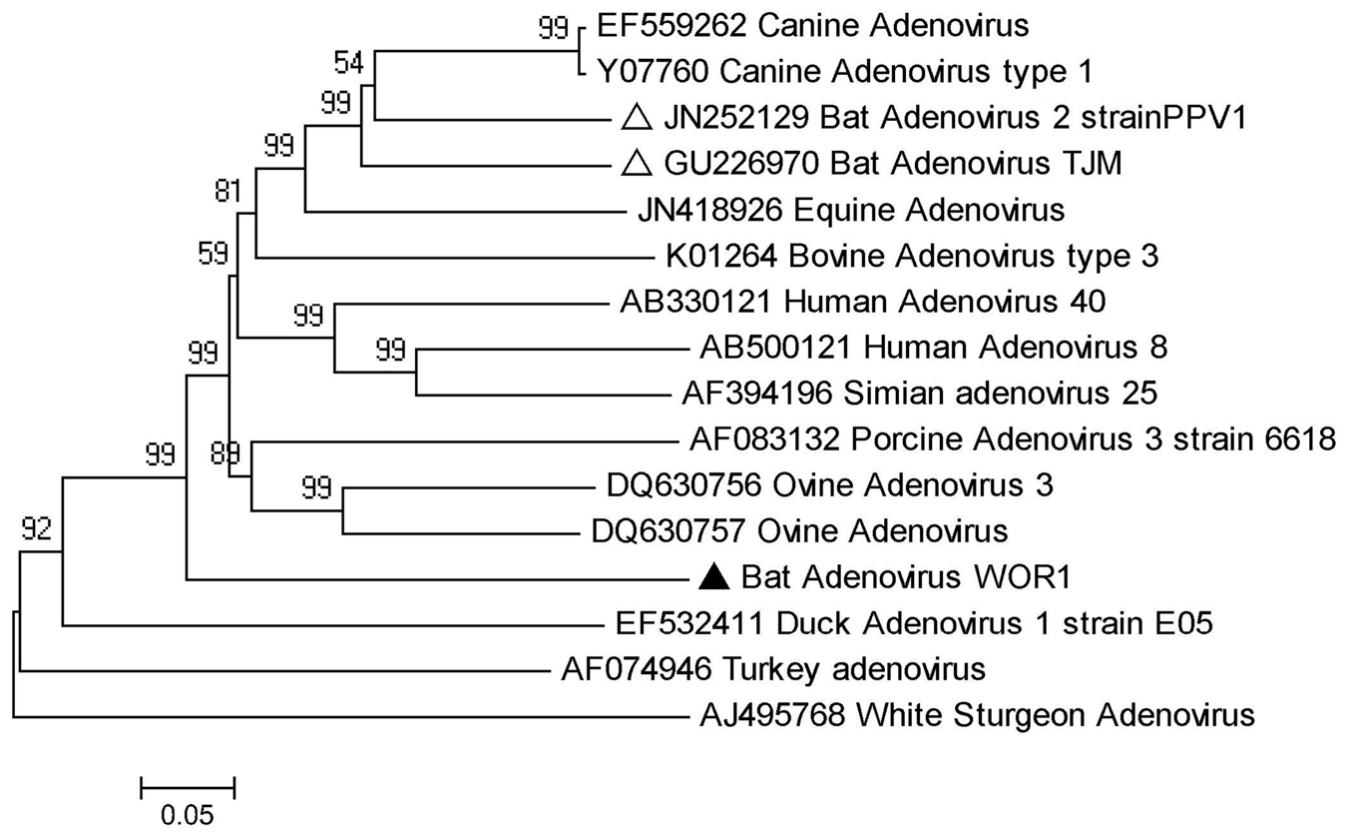
Phylogenetic analysis of bat adenovirus WOR1 and other representatives based on partial hexon aa sequences deduced from 767 bp amplicons. The sequence in our study is identified by a filled triangle and those from previous studies by open triangles.

The members of the family *Adenoviridae* infect a wide range of vertebrates including mammals, birds, amphibians, reptiles and fish, and cause a variety of diseases [51], [52]. In this study, only one contig showing identity with genus *Mastadenovirus* was found in group WO (Table 3). Further screening of all lungs and guts of bat samples by PCR of a partial hexon gene sequence of mammalian adenovirus (767 nt) confirmed that this sequence existed in guts of *R. ferrumequinum* of group WO but none of the other 3 groups. Sequence and phylogenetic analysis showed that this bat adenovirus, given the name WOR1 (accession number: JX863729), is divergent from current mammalian adenoviruses with 40–55% aa identity and with only 55% and 53% aa identities with bat adenovirus TJM strain from Tianjin, China, and bat adenovirus 2 strain PPV1 from Germany respectively (Figure 5) [22], [53]. This indicates that bat adenovirus WOR1 is a new mammalian adenovirus.

### Characterization of Circovirus (CV)

**Figure 6:**
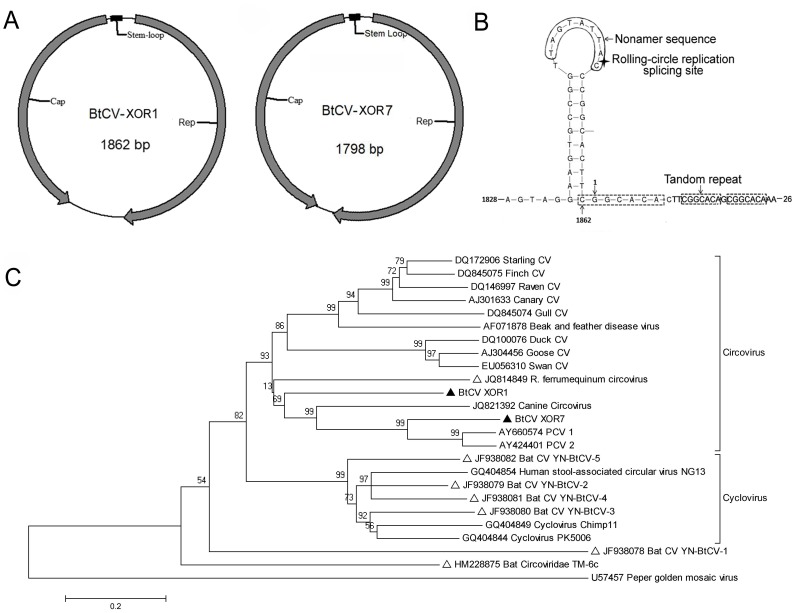
The genome structures of BtCVs in our study and comparative phylogenetic analysis with other CVs. (A) Schematic genome structures of BtCVs; (B) The intergenic stem-loop structure of BtCV XOR1; (C) Phylogenetic analysis of BtCVs based on the complete aa sequence of Rep protein with other representatives. The sequences in our study are identified by filled triangles, those from previous studies by open triangles.

CVs contain a circular genome of 1,700–2,000 nt length which encodes a replication-associated protein (Rep) and a capsid protein (Cap) in opposing directions [54], [55]. CVs were first discovered in American bats in 2010 and in Chinese bats in 2011 [9], [37]. In the present study, only group XO contained CV contigs (Table 3). Further PCR confirmed this result and found that 7% (6/92) of guts of *R. ferrumequinum* in group XO were circovirus positive. Two complete genomes (BtCV XOR1, BtCV XOR7; accession numbers JX863737 and KC339249 respectively) were amplified by inverse PCR. Sequencing revealed that BtCV XOR1 and XOR7 DNAs were respectively 1,862 nt and 1798 nt in length and contained two ORFs in opposite strands (Figure 6A, Table 4). As in other CVs, the two bat CVs had a stem-loop structure between the two ORFs with nonamer motif TAGTATTAC and TAGTATTAC, identical to that of canine CV strain NY214 (JQ821392), beak and feather disease virus, and finch CV (Figure 6B) (23,32). Moreover, three tandem copies of CGGCACA of BtCV XOR1, thought to bind the replicase when the viral DNA starts to replicate by the rolling-circle method, were present at nt 1862-26 with the highest identity to porcine CV-1 and -2 (PCV-1 and PCV-2). The tandem copies in BtCV XOR7, however, were shorter, consisting of the hexamer CGGCAG (Table 4).

**Table 4:** Genomic features of BtCVs and other circoviruses^a^.

Virus	GenomeSize (nt)	Rep (aa)	Cap (aa)	5′ intergenicregion (nt)	3′ intergenicregion (nt)	Nonamer motif	Tandem repeat
BtCV XOR1	1862	295	238	86	171	TAGTATTAG	CGGCACA
BtCV XOR7	1798	313	243	86	39	TAGTATTAC	CGGCAG
RfCV	1760	294	218	90	128	ATAGTATTA	GTGCCGGCC
YN-BtCV-3	1743	277	223	236	2	TAATACTAT	AGTCGCGG
YN-BtCV-4	1741	279	223	229	1	TAATACTAT	ACGAAGTGGACGG
CaCV	1952	290	250	77	249	CAGTATTAC	GGAGCCAC
PCV1	1759	312	233	82	36	TAGTATTAC	CGGCAGC
PCV 2	1768	314	233	83	38	AAGTATTAC	CGGCAGCACCTC

a: RfCV: *R. ferrumequinum* circovirus; YN-BtCV: Yunnan bat circovirus; CaCV: Canine circovirus; PCV: Porcine circovirus.

Phylogenetic analysis based on Rep amino acids and multiple alignments showed that the two BtCVs shared 44% aa identity, indicating they were different isolates. The highest circovirus identities BtCV XOR1 and BtCV XOR7 shared were only 56% with canine CV strain NY214 identified in New York dogs in 2012 and 70% to PCV-1 [56] (Figure 6C).

### PCR Amplification of Bat Hepadnavirus

**Figure 7:**
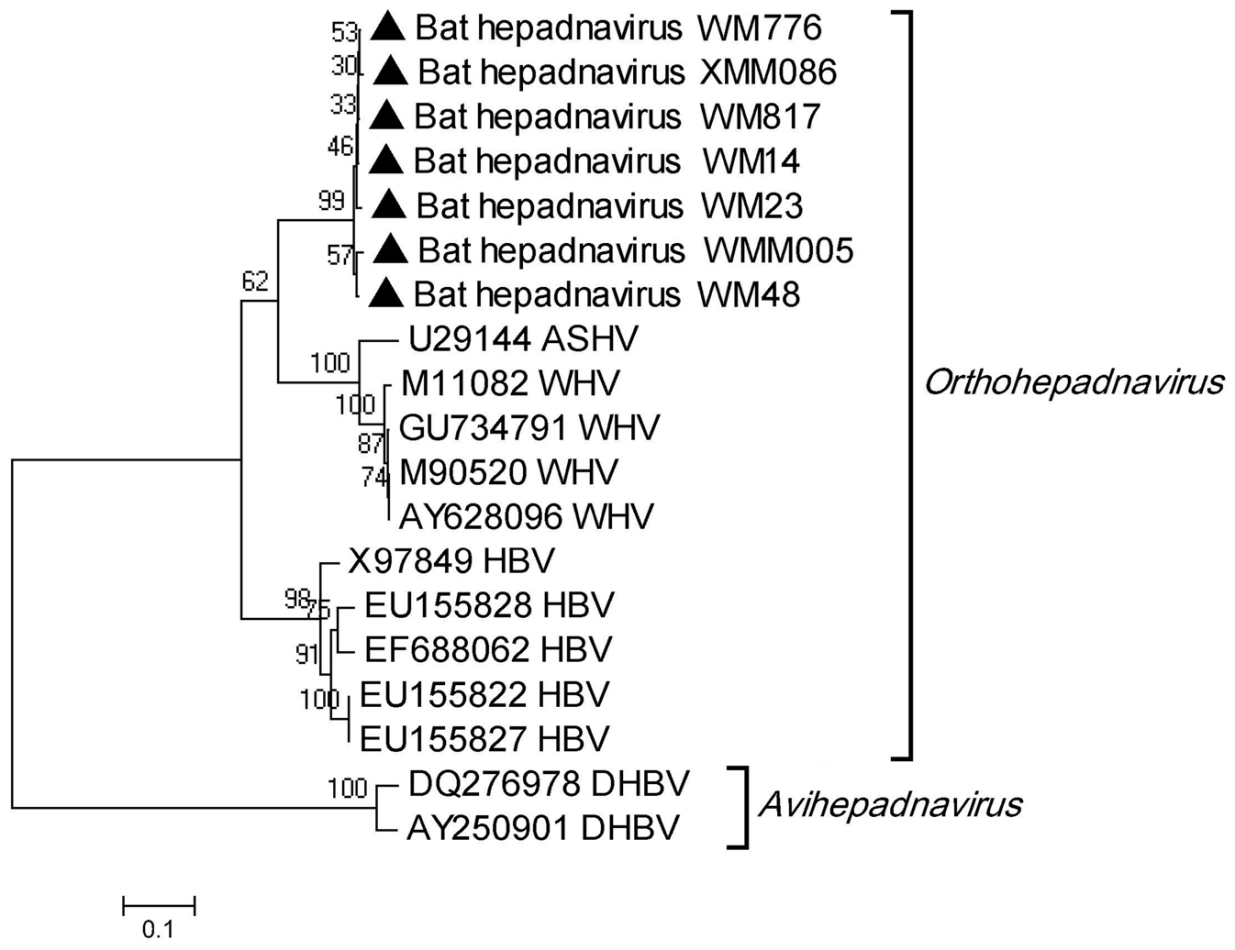
Phylogenetic analysis of bat hepadnaviruses based on 423 bp amplicons of partial S gene, with other representatives of HBV, rodent hepatitis viruses and duck hepatitis B virus. The sequences in our study are identified by open triangles.

Members of family *Hepadnaviridae* have a compact circular genome and comprise two genera, *Orthohepadnavirus* and *Avihepadnavirus*, the former infecting several mammalian species including humans, woodchucks, and ground squirrels [48]. In our study, >10,000 hepadnavirus-like contigs were detected in all four groups through viral metagenomics, showing <70% nt identity with woodchuck hepatitis virus (WHV) and HBV (Table 3). The 423 bp amplicons of partial S genes of 7 selected bat hepadnaviruses showed >97% nucleotide identity with each other, 77% nucleotide identity with WHV and 74% with HBV (Figure 7), indicating that the bat hepadnavirus found in the study is likely a new orthohepadnavirus.

## Discussion

Bats are the natural host of a large variety of mammalian viruses and play an important role in the transmission of many emerging or re-emerging viral diseases to humans and animals from their natural habitats. By 2007, more than 60 bat-borne viruses had been identified globally by traditional virologic methods. This required several decades and the work of numerous laboratories [2], [57]. With metagenomic analysis based on next generation sequencing and high throughput screening, the frequency of discovering new bat viruses has rapidly increased and more than 20 new mammalian viruses or distantly related variants of known mammalian viruses, along with a number of unknown viruses, have been identified in last two or three years by a few laboratories. In addition, the virome constitution of bats has been elucidated by this modern technology [10], [35]–[37]. Using this tool, the present study has revealed a virome of Myanmar bats, comprising 24 viral families capable of infecting vertebrates, insects, plants or bacteria. Sequence comparisons here have uncovered new viruses, further expanding the range of virus composition in the bat population.

In our study, eukaryotes and bacteria respectively accounted for only 2% and 1% of the contigs, showing that the nuclease treatment was effective in the digestion of non-viral nucleic acids. Unknown sequences constituted a large part (95%) of the total with no homologs in GenBank (Table 2).This compares with 91%, 99%, 66% and 51% for unknown sequences reported in, respectively, human feces, infant feces and bat guano [37], [58]. Comparison is difficult, however, since sequence similarities are identified by BLAST search of the Genbank databases, which are continually expanding [58]. Our results showed that about 2% (26,698/1,649,512) of total contigs were virus-related sequences. However, in previous viromes, the proportions of virus-related sequences in total reads or contigs ranged from 0.1%–58% [10], [35]–[37]. Of note is that 45% of viral contigs in our virome classified to vertebrate viruses, while insect viruses and phages constituted only 28% and 27% of viral contigs respectively (Table 3). This compares with less than 10% of total viral contigs related to vertebrate viruses in previous viral metagenomes of bats [10], [35]–[37]. In addition, among 24 viral families identified here, 54% (13 families) were mammalian viruses, much higher and broader than the rates reported in previous virome studies. This difference may be ascribed to the sample type: various organ tissue samples of all bats, along with intestine contents, were used in our study, while the samples used in previous studies were only feces or feces plus oral and pharyngeal swabs [10], [35]–[37]. Mammalian viruses require replication in host cells, with some, but not all, being excreted into the environment through the fecal and/or oral routes. Moreover, excretion of many viruses is intermittent, and therefore the virome obtained from excreted or secreted materials may not represent the complete viral complement of the host. Consequently, mixed tissue samples are likely to provide a more complete virome profile. For instance, since hepadnaviruses are strictly blood-borne viruses and normally not secreted through the fecal and oral routes, this could be the reason why previous metagenomic studies have not found *Hepadnaviridae* in any bat species. In the present study, sampling of bat livers may have permitted successful identification of a bat hepadnavirus. The fact that this constituted the largest population of mammalian viral sequences (Table 3) additionally indicates that bats are potential reservoirs of hepadnaviruses.

Bat viromes have been studied in 3 American states and 10 Chinese provinces [10], [35]–[37]. The present study has provided preliminary data to reveal the bat virome in Myanmar, a country sharing a long border with Yunnan, China, with abundant and diverse bat species. Overall, the bats in Wutao carried 23 of the total identified 24 viral families, with only the absence of family *Circoviridae*, while bats in Sedon carried only 17 families (Table 3), indicating the diversity of the virome in the two counties.

To verify the metagenomic analysis and further understand the viral constitution as well as the prevalence rate in the virome, each of the 853 bat samples was subjected to PCR or RT-PCR for 6 selected viruses (Table 1). Results were completely consistent with that of the metagenomic analysis (Table 3). Coronaviruses have been found to have a wide global geo-distribution [23], [59], [60] and have been identified in previous bat viromes [10], [35]–[37], but were not found in the present study. In confirmation of this, none of the bat samples tested positive for coronavirus by RT-PCR screening using published method [60], this may be due to insufficient bat sampling and limited locations.

While PCR and RT-PCR results showed that *M. fuliginosus* and *R. ferrumequinum* were the only species to harbor the selected viruses, this difference was possibly due to insufficient sampling of the other four bat species. The 2 positive bat species further showed differences in viral constitution with *M. fuliginosus* harboring Astv, Ifv, hepadnavirus and BoV, while *R. ferrumequinum* harbored Astv, CV, Adv and AVV, indicating the existence of co-infection with multiple viruses in both species. In addition, even the same species had a different viral constitution in different locations. For example, *M. fuliginosus* harbored astrovirus in Sedon, but not in Wutao; *R. ferrumequinum* harbored only CV in Sedon, but Astv, Adv and AVV in Wutao. These data show that bat virome varies among species and geo-locations, indicating that a complex viral ecology exists in the bat population. Similar differences in the bat virome have also been reported in previous studies [10], [35]–[37], and therefore huge diversities in bat virome in the world can be expected.

Results of PCR and RT-PCR screening also revealed the prevalence rates of selected viruses in the bat virome (Table 1), which were not investigated in previous studies [10], [35]–[37]. Astv, AAV, Adv and CV sequences identified here in Myanmar bats have also been noted in the previous viromes in North America and China, indicating that they are common virus species in bats with wide geographic distribution. For example, three species within the genus *Rhinolophus* (*R. pusillus*, *R. luctus*, *R. ferrumequinum*) in China have been found to harbor CVs [10], [35], as in the present study. In addition, *Miniopterus schreibersii* and *Rhinolophus sinicus* were found to harbor Astv in China [13], a virus shown in our study to be present in *M. fuliginosus* and *R. ferrumequinum* in Myanmar.

Since the whole digestive tract from stomach to anus, together with the gut content, was pooled with other organs in the sample preparation, the identification of insect viruses in the bat tissue samples was not surprising and simply reflected dietary traits [37]. In our virome study, contigs of *Ectropis obliqua* and *Perina nuda* insect viruses were detected only in *M. fuliginosus* of both XM and WM groups, suggesting that these bats frequently prey on these insects. The most likely explanation for the presence of plant viruses such as tospovirus and cucumovirus in some samples is that the bats prey on insects that feed on plants. Presence of plant viruses may therefore simply reflect the natural food chains. Plant viruses have also been detected in bat guano in North America and China [35]–[37].

As in previous metagenomic analyses [10], [35]–[37], many novel mammalian viruses have been identified in the present virome, including Astv, CV, Adv, AVV, and bat hepadnavirus. Members of the family *Hepadnaviridae* have a narrow host range and can infect rodents, primates and birds, but have never before been reported in bats [48], [61]. In the present study, hepadnavirus-like contigs were the second largest population, and showed a genetic similarity to WHV and HBV. PCR and sequencing of a partial S gene sequence showed that the bat hepadnavirus identified here had approximately equal identity with WHV and HBV, strongly suggesting that it should be classified as a new species in genus *Orthohepadnavirus*.

The family *Circoviridae* has two genera, *Circovirus* and *Gyrovirus*, and a wide host range from mammalian animals to avian species [48], [54], [55], [62]. CVs are important pathogens of pigs and have a huge impact on the pig industry. These viruses have also been found in some avian species causing immumosuppression and growth retardation [62]–[65]. However, a viral metagenomics analysis of bat guano in North America first identified their presence in bats [37]. Since then, CVs have been commonly found in bats and have shown a large genetic diversity [9], [10], [35]. According to ICTV criteria, BtCV XOR1, observed in this study, should be a new species since it shares <60% aa identity with known circoviruses [48]. Topologically, the known bat circoviruses are grouped into genera *Circovirus* and *Cyclovirus*, with some having higher similarities to other animal circoviruses than to bat circoviruses (Figure 6C). For example, BtCV XOR1 in our study shared the highest (56% aa) identity with canine CV (No.JQ821392), and only 48% identity with BtCV YN-BtCV-2 (No. JF938079). The large genomic diversity of bat circoviruses suggests that co-evolution of the viruses could occur in bats.

There are currently the only two members in the genus *Bocavirus* and share 43% sequence identity [47], [66]. Animal bocaviruses were first identified in the 1960s, and can cause respiratory, gastrointestinal and reproductive diseases [66]. Human bocavirus was first reported in Sweden in 2005, then in China, 2006, and has been associated with upper and lower respiratory infection and gastroenteritis worldwide [67]–[69]. Wu et al. first reported their presence in *M. myotis* in 2012 [10]. Our study expands their range to include *M. fuliginosus* in Myanmar. Sequence analysis showed that the Myanmar isolate had about 53% aa identity with canine minute virus (the highest identity) and only 43% identity with *Myotis myotis* bocavirus. Full genome alignments also revealed their divergences. According to the ICTV criterion that a bocavirus with <95% nt identity in its NS gene can be defined as a new species, Bt BoV XM30 and WM40 likely form a new species within the genus *Bocavirus* [48].

Members of family *Astroviridae*, comprising genera *Avastrovirus* and *Mamastrovirus*, are important pathogens and usually cause acute gastroenteritis of human and a broad spectrum of animals, including livestock, poultry, companion animals, fur animals and marine mammals [48], [70]–[72]. The genetically diverse mamastroviruses consisted of 6 species in 2009 [73], but since the first bat Astv report in 2008 [12] identification of numerous new Astvs had expanded the list to 19 species by 2011, with 7 relating to bats [48]. They have been identified in several bat species including *Miniopterus schreibersii, Scotophilus kuhlii, and Rousettus leschenaultia* in Hainan, Hunan, Anhui, Guangxi and Fujian provinces, China [12]–[14], and from *M. myotis* in Germany [59]. In the present study, more novel bat Astvs were detected that diverged from the current 19 Astv species, as shown in figure 1, indicating that these form new species within the genus *Mamastrovirus*. The continuing emergence of new mamastrovirus species and their wide genetic divergence in bats indicates that there could still be more unknown bat astroviruses.

In conclusion, the virome of Myanmar bats obtained by Solexa sequencing-based viral metagenomics with specific PCR confirmation, using various tissue samples and gut contents, has identified many new mammalian viruses in these animals. Our results, together with previous studies [10], [35]–[37], show that the composition of bat viromes differs depending on geographical location and bat species. Current bat viromes have therefore uncovered only the tip of the iceberg as regards virus flora present in these mammals, and future studies involving a wider sampling of bat species in different locations will undoubtedly increase our understanding of the global diversity of bat viruses. However, most of the new viruses found in bats by metagenomic analysis have very low identity with known viruses and their direct transmission to cause diseases of humans and domestic animals is unknown. To determine the tropism restrictions and pathogenicity of these new mammalian viruses, routine virological approaches, such viral isolation and characterization by tissue culture and experimental infection, will still be necessary.
