# Correction: Virome Profiling of Bats from Myanmar by Metagenomic Analysis of Tissue Samples Reveals More Novel Mammalian Viruses

**DOI:** 10.1371/annotation/68f77773-a2a0-4bfe-b5e6-950dc30b79f9

**Published:** 2013-06-28

**Authors:** Biao He, Zuosheng Li, Fanli Yang, Junfeng Zheng, Ye Feng, Huancheng Guo, Yingying Li, Yiyin Wang, Nan Su, Fuqiang Zhang, Quanshui Fan, Changchun Tu

The second accession number given in the first sentence of the section "Nucleotide Sequence Accession Numbers" in Materials and Methods is incorrect. The correct accession number is: SRA059295. 

